# Enhancing Undergraduate Interest and Preparation for Research in Aging and Neurologic Diseases

**DOI:** 10.1093/geroni/igaf122.3926

**Published:** 2025-12-31

**Authors:** Natalie Galucia, Brian Carpenter, Claire Wininger, Michele Dinman

**Affiliations:** Washington University in St. Louis, Saint Louis, Missouri, United States; Washington University in St. Louis, Saint Louis, Missouri, United States; Washington University in St. Louis, Saint Louis, Missouri, United States; Washington University in St. Louis, Saint Louis, Missouri, United States

## Abstract

Many students pursuing careers in research, policy, or practice in the social and health sciences overlook gerontology and geriatrics due to limited exposure to aging. Additionally, negative stereotypes about older adults and aging discourage students from pursuing careers in these fields. Our NINDS-sponsored summer research program aims to meet this workforce need by recruiting undergraduate trainees with varied academic backgrounds and interests to provide rigorous didactics, structured mentoring, and hands-on research experience. The goal of the program is to foster interest, attitudes, and skills in the aging field and research pertaining to neurologic disorders among older adults. Students spend eight weeks working in research labs under the guidance of faculty mentors and attending professional development seminars. Seven cohorts of participants (n = 88, 68.8% female, 29.1% male, 46.5% White, 19.8% Black, 25.6% Asian) were surveyed before and after the program. Students’ self-reported knowledge of research methods increased 34.5%, and skills in data visualization increased 36.3%. The age they consider someone to be “old” increased from 66 to 73 years, suggesting a reframing of how they view aging. Students also considered aging more relevant to their personal lives. A significant proportion of alumni have enrolled in research-oriented graduate school or medical school (36.8%) and pursued public health- or research-related positions (72.3%). Overall, the program has had a positive impact, increasing knowledge and confidence in conducting research, and encouraging students to consider aging in their personal and professional lives.

